# Development of an EMG-Based Muscle Health Model for Elbow Trauma Patients

**DOI:** 10.3390/s19153309

**Published:** 2019-07-27

**Authors:** Emma Farago, Shrikant Chinchalkar, Daniel J. Lizotte, Ana Luisa Trejos

**Affiliations:** 1 Department of Electrical and Computer Engineering, Western University, London, ON N6A 5B9, Canada; 2 Division of Hand Therapy, Hand and Upper Limb Centre, St. Joseph’s Health Care, London, ON N5V 3A1, Canada; 3 Department of Computer Science, Western University, London, ON N6A 5B9, Canada; 4 Department of Epidemiology & Biostatistics, Western University, London, ON N6A 5B9, Canada; 5 School of Biomedical Engineering, Western University, London, ON N6A 5A5, Canada

**Keywords:** classification, electromyography (EMG), feature selection, rehabilitation, wearable devices

## Abstract

Wearable robotic braces have the potential to improve rehabilitative therapies for patients suffering from musculoskeletal (MSK) conditions. Ideally, a quantitative assessment of health would be incorporated into rehabilitative devices to monitor patient recovery. The purpose of this work is to develop a model to distinguish between the healthy and injured arms of elbow trauma patients based on electromyography (EMG) data. Surface EMG recordings were collected from the healthy and injured limbs of 30 elbow trauma patients while performing 10 upper-limb motions. Forty-two features and five feature sets were extracted from the data. Feature selection was performed to improve the class separation and to reduce the computational complexity of the feature sets. The following classifiers were tested: linear discriminant analysis (LDA), support vector machine (SVM), and random forest (RF). The classifiers were used to distinguish between two levels of health: healthy and injured (50% baseline accuracy rate). Maximum fractal length (MFL), myopulse percentage rate (MYOP), power spectrum ratio (PSR) and spike shape analysis features were identified as the best features for classifying elbow muscle health. A majority vote of the LDA classification models provided a cross-validation accuracy of 82.1%. The work described in this paper indicates that it is possible to discern between healthy and injured limbs of patients with MSK elbow injuries. Further assessment and optimization could improve the consistency and accuracy of the classification models. This work is the first of its kind to identify EMG metrics for muscle health assessment by wearable rehabilitative devices.

## 1. Introduction

Musculoskeletal (MSK) conditions are disorders or injuries that affect the bones, joints, skeletal muscles and/or connective tissues. Without adequate rehabilitation, MSK injuries could become chronic as a result of joint stiffness and reduced muscle strength [1].

The development of lightweight robotic braces offers a potential for improved rehabilitation. Wearable rehabilitation robotic devices have been successful in improving rehabilitation program compliance, accelerating recovery, and monitoring health for patients suffering from neuromuscular damage from stroke [2]. Mechatronic braces for patients with neurological disorders have been developed to assist with mobility and to allow patients to perform exercises at home and at their own convenience [3]. However, there has been little work done to develop rehabilitation devices for patients with MSK injuries, in which damage affects the bones, muscles, and connective tissues, but in which the central nervous system is intact.

The rehabilitation of the elbow following trauma is an inherently challenging process due to the complexity of the elbow joint [4]. Inadequate rehabilitation leads to the development of stiff elbow and a loss of range of motion (ROM) [5]. Rehabilitation following elbow trauma should involve passive and active ROM exercises to ensure that the ensuing collagen remodeling and elongation of the tendinous and capsular tissues allow for joint motion. ROM exercises typically involve elbow flexion and extension, and forearm pronation performed with the elbow at 90 degrees [4].

Most of the scientific evidence to support certain rehabilitation approaches is based on retrospective and case series studies with small sample sizes, not on randomized clinical trials (RCTs) [5], and the optimum dosage of the frequency and repetition of ROM exercises is unknown [4,5]. Furthermore, the rehabilitative procedures tested are often poorly described and not reproducible [5], and the outcome measures often depend on the therapist’s perspective [6]. Measurements from smart wearable devices could improve the objectivity of muscle health assessment, and lead towards stronger evidence-based rehabilitation.

An ideal rehabilitative smart device would be capable of objectively and autonomously determining a patient’s muscle health. This would enable a path towards (1) improved diagnostics, (2) the development of individualized therapies specific to a patient’s level of health, and (3) the identification of objective outcome measures to inform evidence-based rehabilitation practices. Electromyography (EMG), the study of the electrical currents generated during muscle contraction, offers a possible solution for assessing muscle health with a smart brace. EMG signals can be collected directly from muscle units by inserting a needle into the muscle fiber. Surface EMG (sEMG) signals are collected noninvasively by placing electrodes on the surface of the skin. Quantitative EMG analysis is an established diagnostic tool for patients with nerve damage and skeletal muscle damage.

A range of models including artificial neural networks, support vector machines, and decision trees have been used to successfully classify needle EMG signals as neuropathic, myopathic, or normal [7,8]. There is some evidence that sEMG data from patients with elbow injuries conform to patterns that distinguish the level of injury and could therefore be used to quantify the success of a therapy. Patients with elbow motion deficits following injury have been observed to have increased sEMG activity in their elbow muscles during elbow flexion and extension compared to controls [9]. However, there is no classification method that can be implemented in an elbow rehabilitation device to monitor and assess patient health following elbow trauma. If these differences could be modeled using a classification system, they could be implemented in a rehabilitative robotic device and a patient’s health could be monitored as they progress through the rehabilitation process.

As a first step in this direction, this paper investigates methods of using sEMG data for classifying upper limbs as healthy or injured. The remainder of the paper is organized as follows: Section 2 provides background information about feature extraction for sEMG signal classification. Section 3 explains the methods used in the study, including data collection, feature extraction, feature selection, and classification. Section 4 describes the classification results, Section 5 is the discussion, and Section 6 provides the conclusions.

## 2. Background

EMG data are typically classified using pattern recognition techniques [10]. Following EMG acquisition, the data are windowed into segments, and features are extracted from each segment. Feature extraction allows useful information to be obtained from the sEMG signal, and reduces unwanted information and noise [11]. Numerous features have been proposed for sEMG classification [12,13]. The features used in this study are summarized in Table 1.

### 2.1. sEMG Features

sEMG features can be categorized by the type of information about the signal that they provide [13]. The following categories of features are commonly used:

#### Time Domain Features

Features extracted from the time domain can provide information about the (1) energy, (2) frequency, and (3) information complexity of the sEMG signal. The mean absolute value (MAV), Willison amplitude (WAMP), and wavelength (WL) features were found to provide the best motion classification accuracies of these three subclasses of features [13]. Time domain features can be further derived from features calculated for each window segment. For example, the slope of the mean absolute value (MAVS) feature is the difference of the MAV between two adjacent window segments. Time domain features are preferable for real-time devices because they require low computational complexity to calculate [13].

The Hudgins feature set, developed by Hudgins in 1993, consists of the following five time domain features: MAV, MAVS, WL, slope sign change (SSC), and zero crossings (ZC) [18]. MAVS is typically omitted from the Hudgins set in the most recent literature [20]. Many myoelectric devices implement the Hudgins feature set or a variation of the Hudgins feature set because it includes features that are effective for motion classification and computationally simple to extract [20].

#### Frequency Domain Features

Frequency domain features are primarily used to study muscle fatigue and motor unit recruitment. They are not as computationally efficient as time domain features and provide weaker performance for motion classification [13,15].

#### Spike Shape Analysis Features

Further information about muscle activation and motor unit activity can be assessed from the morphology of the sEMG signal with spike shape analysis (SSA) features [21]. A spike is defined as a single upward and downward deflection that is greater than a predefined threshold amplitude. The threshold is typically the 95% confidence interval for baseline EMG activity [14].

#### Prediction Model Coefficients

The fourth-order autoregressive (AR) and cepstral models are commonly implemented for sEMG signals [13]. The second order AR coefficients (AR2) have also been successful for motion classification when combined with the root mean square (RMS) time domain feature [15].

#### Entropy Features

The approximate entropy (ApEn) feature is a measure of system complexity used to classify stochastic processes [16]. ApEn has been applied to sEMG signals for motion classification [22]. ApEn represents the likelihood that similar patterns of observations will not be followed by similar observations. The sample entropy (SampleEn) feature is a refinement of ApEn that improves the consistency of comparisons between data sets [19].

#### Fractal Dimension Features

Fractal dimension (FD) features provide information about the morphology, spectrum, and variance of the EMG signal. The FD is a measurement of the non-linear property of a signal, and is related to muscle size and complexity, but is unrelated to the strength of muscle contraction. FD is useful for classifying motions using single EMG channels, and low-level muscle activations [17]. Several FD features have been proposed for the classification of EMG signals, including Higuchi’s fractal dimension (HFD), maximum fractal length (MFL), and detrended fluctuation analysis (DFA).

#### Higher Order Statistics

Higher order statistics of EMG signals, such as skewness (SKEW) and kurtosis (KURT) can identify details of the EMG signal that are missed when the signal is assumed to be a Gaussian process. For example, the kurtosis of the EMG signal tends to be greater than zero, and decreases towards approximating a Gaussian distribution as the contraction level increases [23].

### 2.2. Features for Evaluating Muscle Health

Several sEMG features have been found to exhibit differences between healthy subjects and patients with neuromuscular or muscle disorders including Duchenne muscular dystrophy [24], non-specific arm pain [21], stiff elbow [9], and elbow trauma [25].

Spike shape analysis was implemented for identifying and evaluating patients of non-specific arm pain. Significant increases in mean spike amplitude (MSA), mean spike frequency (MSF), mean spike slope (MSS), and mean number of peaks per spike (MNPPS) and significant decreases in mean spike duration (MSD) were observed in the sEMG signals collected from the extensor carpi radialis muscle in subjects with non-specific arm pain compared to controls [21].

Haddara [25] compared six sEMG features (median frequency (MDF), mean frequency (MNF), zero crossings (ZC), RMS, MAV, and MSA) collected from elbow trauma patients and a group of healthy subjects. Statistically significant differences were primarily identified using the RMS and MAV features. The RMS and MAV features collected from the patients at the end of the therapy program were found to resemble the healthy population more closely. The frequency domain features, MDF and MNF, showed no significant differences between the groups.

### 2.3. Classification

Following feature extraction, machine learning classifiers (including linear discriminant analysis (LDA), support vector machines (SVM), and decision tree classifiers) are applied to the extracted features to classify the EMG data. LDA is a robust classifier and is advantageous for embedded processors involved with real-time applications because it provides fast prediction speeds and small memory usages. LDA has been applied to a variety of EMG classification problems [26], and is generally found to provide acceptable classification accuracies [20]. An extension of the LDA classification method is the SVM classifier, which uses separating hyperplanes to distinguish between two classes of data. The SVM classifier has been used for many EMG applications including motion classification [15] and the diagnosis of neuromuscular disorders [27].

LDA and linear SVM models may perform poorly if the relationships between features are non-linear. Decision tree classifiers are simple models that provide easily interpretable results, and can outperform linear models when classifying non-linear data. A single decision tree classifier determines an outcome based on a series of splitting rules starting at the top of a tree and continuing into a series of branches. The decision tree stratifies the feature space into regions to provide the prediction. A single decision tree model is susceptible to over-fitting and a lack of robustness. These problems can be avoided by aggregating many decision trees. The random forest (RF) algorithm prevents decision tree models from considering most of the available predictors at each split [28].

### 2.4. Summary

Pattern recognition techniques have been applied to the classification of sEMG signals by motion [13,29], force [30], neuromuscular health [7], and fatigue [31,32]. There has not been a study, however, classifying muscular health of patients following MSK injury, although preliminary evidence suggests that muscle activation patterns differ between healthy and injured patients [25]. This paper investigates the application of sEMG features to the classification of muscle health following MSK injury.

## 3. Methods

Working from the sEMG features presented above, this section now describes the methods of data collection and data analysis (feature extraction, feature selection, classification, evaluation, and optimization) with the objective of identifying and assessing the ability of features to distinguish elbow muscle health.

### 3.1. Data Collection

Data collection began following approval from the Health Science Research Ethics Board at Western University (Reference No. 106913, approved on 7 February 2017). Thirty patients (21 male, 9 female) with traumatic elbow injuries were recruited from the Roth McFarlane Hand and Upper Limb Centre at St. Joseph’s Hospital in London, Ontario. Patients presented with elbow fractures, elbow dislocations, arthroscopic releases, and bicep tendon repairs, including surgical implants. Patients were excluded if they indicated that they had congenital MSK defects, or if they had previously experienced elbow trauma on their contralateral limb. The mean time since the injury was sustained was 9.6 ± 5.9 weeks. The mean reported age of the patients was 45.0 ± 11.5 years, the mean reported height was 175 ± 9.8 cm and the mean reported weight was 89.2 ± 20.3 kg. Seventeen subjects had injured their dominant hand and 13 had injured their non-dominant hand.

sEMG signals were recorded from the following 7 muscles: biceps brachii (BB), triceps brachii lateral head (TBlat), triceps brachii long head (TBlong), pronator teres (PT), brachioradialis (BRD), extensor carpi ulnaris (ECU), and flexor carpi ulnaris (FCU). Electrodes were placed according to the SENIAM recommendations for electrode placement [33].

All sEMG signals were collected and amplified with a commercial wireless myoelectric system (Trigno Wireless system, Delsys Inc., Natick, MA, USA). The signals were amplified with a gain of 300×, and the sampling frequency was 1925.93 Hz. The sensors were affixed to the skin using the recommended double-sided adhesive stickers (Trigno Sensor Skin Interface SC–F03).

**Figure 1:**
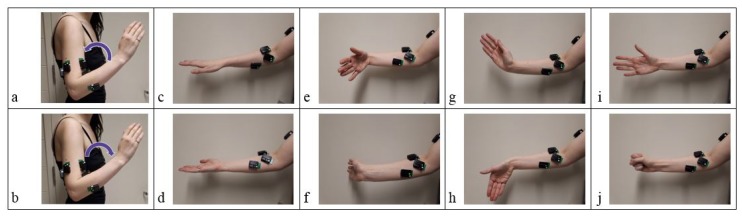
Ten upper-limb motions performed: (**a**) elbow flexion (EF), (**b**) elbow extension (EE), (**c**) forearm pronation (P), (**d**) forearm supination (S), (**e**) wrist flexion (WF), (**f**) wrist extension (WE), (**g**) ulnar deviation (UD), (**h**) radial deviation (RD), (**i**) hand open (HO), and (**j**) hand close (HC).

Patients were asked to perform three repetitions of the following motions with both the injured and the contralateral uninjured arm: elbow flexion (EF), elbow extension (EE), forearm pronation (P), forearm supination (S), wrist flexion (WF), wrist extension (WE), ulnar deviation (UD), radial deviation (RD), hand open (HO), and hand close (HC). Motions were selected based on standard elbow rehabilitation exercises. Wrist and hand exercises were included because elbow trauma patients are also encouraged to perform wrist and finger exercises during rehabilitation [4]. The forearm, wrist, and hand exercises were performed with the elbow held at approximately 90 degrees (Figure 1).

Motions were performed in sets with the patient pausing at the end of each motion. The following order of motion sets was used for every trial: EF/EE, P/S, WF/WE, UD/RD, and HO/HC. Each motion set was performed with the injured arm three times, and then with the uninjured arm three times. The forearm, wrist, and hand exercises were performed with the elbow at approximately 90 degrees. The patients were instructed to perform all motions at a comfortable pace.

### 3.2. Data Processing

All off-line data analysis was performed using MATLAB software (The MathWorks Inc., Natick, MA, USA, Version R2017b).

The sEMG data were divided into segments representing each motion based on muscle activation. The double-threshold method for detecting EMG onset was used to facilitate segmentation. The double-threshold method detects a muscle activation onset once a certain number of consecutive samples, th2, exceeds a threshold amplitude, th1 [34]. The th1 value was $\bar{b}+15\sigma$, where *b* is the baseline value of the sEMG signal and σ is the standard deviation of *b*. The th2 value was set to 25. The signals were first conditioned with the Teager-Kaiser energy operator (TKEO) and then rectified and passed through a 2nd order Butterworth 50 Hz low pass filter to improve the robustness and accuracy of muscle activation onset detection [35].

The TKEO is defined as follows [35]:

(1) $$\Psi \left[x\left(n\right)\right]=x^{2}\left(n\right)-x\left(n+1\right)x\left(n-1\right)$$

where *x* is the EMG value, and *n* is the sample number.

The segmentations for each sEMG recording were verified visually and sets that were not segmented correctly by the algorithm were segmented manually. About 50% of the data sets had to be resegmented manually. Data sets from three subjects were excluded from further analysis after visual inspection indicated that the data were corrupted.

The sEMG signals were filtered with a 2nd order Butterworth 20–400 Hz band pass filter to remove low frequency motion artifacts, and uninformative high-frequency components. The signals were also filtered with a 60 Hz notch filter to reduce power line interference [36].

### 3.3. Feature Extraction

Forty-two EMG features were extracted from each EMG segment. These features are listed in Table 1. The features were selected to be representative of each type of information that has been extracted from sEMG signals (as described in Section 2). These features were also used to develop three preliminary feature sets, as follows:

- Feature Set 1 (FS1): MAV, SSC, WL, ZC
- Feature Set 2 (FS2): RMS, AR2
- Feature Set 3 (FS3): MSA, MSF, MSS, MNPPS, MSD

FS1 is the Hudgins feature set [18]. FS2 is the feature set developed by Oskeoi and Hu [15] that was observed to perform well for motion classification. FS3 is a feature set consisting of spike shape features [14]. All features were calculated from the signal collected over the entire motion. One feature was obtained for each muscle. For example, for FS1 there were 4 features times 7 sEMG muscle channels for a total of 28 features in the feature vector for each segment. Feature values for healthy and injured limbs from the same patient were calculated independently. The features were not adjusted so that direct comparisons could be made between the healthy and injured limbs of individual patients.

### 3.4. Classification

Classification models were developed and evaluated for each of the ten motions separately. The LDA, SVM, and RF classification models were investigated. The LDA classifier was selected because it is simple, and has been found to be effective for classifying EMG signals in the literature. The SVM classifier was selected as an extension of the LDA classifier. The RF classifier was selected due to its usefulness for classifying stroke rehabilitation outcomes [28]. The RF classifier was generated from 200 decision trees. Classification models were initially developed to distinguish between healthy and injured limbs. The classification models were also investigated for distinguishing between patients at two different stages of rehabilitation: 0–6 weeks and 7+ weeks.

### 3.5. Evaluation

The classification accuracies for the feature sets extracted from each motion were evaluated for the LDA, SVM, and RF models. The classification accuracies were computed using a leave-one-patient-out cross-validation method. One patient was used as a test set, and the remaining patients were used as the training set. This process was repeated for each patient. The accuracy was calculated as the number of correct classifications divided by the total number of patients.

### 3.6. Optimization of Feature Sets and Models

Both the feature sets and classification models were further optimized. A majority vote was performed for each patient to combine the outputs of the individual motion models. The majority vote models were further optimized by generating weighted majority vote models.

Feature selection algorithms are used to choose the best features in order to improve the classification accuracy and to minimize the number of features required for classification. A feature set should maximize the class separability within a feature space, so that classes are maximally distinguishable. The computational complexity of a feature set should be kept as low as possible to reduce the feature extraction time and the hardware memory requirements [37].

The RELIEFF feature selection algorithm [38] provides a weight for each feature based on its predictive ability. The algorithm iterates through instances of each feature and searches for the *k*-nearest neighbours in the same class (nearest hits) and from a different class (nearest misses). A good feature has a similar value to the nearest hit classes, and a very different value from the nearest miss classes. The differences between each feature instance and the nearest hits are added to the feature weight, and the differences between each feature instance and the nearest misses are subtracted from the feature weight. Feature weights are scaled on the interval [−1,1]. The best individual features were found by comparing their individual performance in a majority vote model. The RELIEFF algorithm [38] was used with the number of *k*-nearest neighbours to search for set to 10, as recommended in [28] to search for the best combinations of features within a feature set.

Following the development and testing of the various classification models, the influence of patient characteristics (sex, age, body mass index (BMI), and the time since injury) on the outcomes of the models was investigated. Age was divided into three categories: (1) <30 (n=8), (2) 30–45 (n=13), (3) >45 (n=6). BMI was divided into categories of normal (18.5–25), overweight (25–30), and obese (>30). Each BMI category contained nine patients. Three categories of time since injury were investigated: healthy, the early stages of rehabilitation (0–6 weeks of therapy), and the late stages of rehabilitation (7+ weeks of therapy). The rationale behind these divisions was that strengthening rehabilitation exercises begin at 7–8 weeks of therapy [4,5]. As well, patients in later stages of recovery have been observed to have more similar EMG metrics to healthy subjects [25]. The patient characteristics were input into the classification models as non-zero ordinal categories, and the models were reevaluated.

## 4. Results

### 4.1. Preliminary Feature Sets

The three preliminary feature sets (FS1–FS3) were developed based on feature set recommendations in the literature. Classification models were developed for each individual motion. The classification results for each feature set and motion are shown in Table 2.

The models distinguished between the two levels of health with accuracies ranging from 45.9–79.6%, depending on the classification algorithm and the motion. The RF models provided the best classification accuracies for the majority of the motions when used with FS1 and FS2. For example, the RF classification accuracies for FS1 ranged from 56.8–72.2%, while the LDA and SVM classification accuracies ranged from 55.6–69.1% and 54.9–67.9%, respectively. Better performance for the non-linear RF classifier suggest that many of the relationships between the features in FS1 and FS2 that influence health are non-linear. There was no evident best classifier for FS3, although the best accuracy was obtained with the LDA model for WF, which provided an accuracy of 79.6%.

The initial classification results suggest that some motions are better than others for classifying patient health. Table 3 shows the range of classification accuracies for each motion. The EE motion provided the range with the highest accuracies overall (62.3–77.8%). The models for the WE and HO motions provided the ranges with the lowest classification accuracies (54.3–66.7% and 48.2–64.2% respectively). All other motions achieved a classification accuracy of at least 72.2% for one of the models; however, the RD motion provided classification accuracies below 70% with the exception of only one classification model.

### 4.2. Individual Features

Following the previous analysis, the performance of each feature were evaluated individually. A majority vote was taken across the ten different motions for each individual feature. The decision agreed upon by the majority of the ten individual motion models for each patient was selected as the final classification result. This procedure reduced the effect of errors made by individual motion models. The majority vote models were evaluated using a leave-one-patient-out cross-validation. Table 4 shows the individual classification performance ranked in order of LDA classification performance.

The individual feature models classified between healthy and injured limbs with accuracies ranging from 46.3–76.5%. The LDA classifier provided the highest classification accuracy for 27/41 of the individual features, therefore the following features were ranked the highest for each feature category: LOG (time domain: energy), DASDV (time domain: information complexity), MYOP (time domain: frequency), MAVS (time domain: multi-window), PSR (frequency domain), MSD (spike shape analysis), AR4 (prediction model coefficients), ApEn (entropy), MFL (fractal dimension), and SKEW (higher order statistics).

### 4.3. Feature Set Development

The individual feature performances were used to inform the development of new feature sets. FS4 consisted of the overall top ranked features. The MFL and MYOP features were selected because adding subsequent features was found to degrade the classification accuracy. FS5 was developed to include the maximum ranked feature within each feature category. SKEW was excluded because of its low individual performance (below 60% for all classifiers). FS5 ultimately consisted of the following features: LOG, DASDV, MYOP, MAVS, PSR, AR4, ApEn, MFL, MSD.

Feature reduction is necessary in order to improve the performance, speed, and memory usage of the classifiers. FS5 contained nine features, therefore it was desirable to minimize the number of features in this set. The RELIEFF algorithm was implemented to rank the top scoring features in FS5. The MSD, PSR, and MFL features were consistently ranked among the best features in FS5 for all motions, and were selected for the optimized feature set. This method would likely lead to upward bias, therefore future work could entail reevaluating the selected features on a new set of patient data.

The classification accuracies for the new feature sets are summarized in Table 5. FS4 provided the best ranges of classification accuracies when used with the LDA and SVM classifiers (63.0–78.4% and 60.5–79.6%); however the ranges achieved with the RF classifier were poor (57.4–70.4%). FS5 tended to work better with the SVM and RF classifiers, and tended to have poor classification accuracies when used with the LDA classifier. Following optimization with the RELIEFF algorithm, FS5 tended to achieve higher classification accuracies, although the accuracies were degraded for some of the motions and classifiers. The LDA classifier demonstrated the greatest improvement following feature reduction. The RF classification results did not improve following the feature reduction.

### 4.4. Majority Vote Models

Majority vote models were developed for all of the feature sets. Majority vote decisions for each patient were obtained using the outputs from all of the motion models, and from the outputs of only the top motion models (EF, EE, P, S, WF, UD, and HC). Table 6 shows the majority vote classification accuracies.

The classification accuracies ranged from 58.6–74.7%. Most of the accuracies obtained with the majority vote models were within the upper range of accuracies that had been achieved with the individual motion models. Two of the majority vote models (SVM with FS1 and LDA with FS2) surpassed the accuracies of all of the individual motion models. This indicates that misclassification can be reduced by performing a majority vote, although the motion models often still agree on incorrect classifications.

The majority vote model was extended by implementing a majority vote decision between “top” motions that provided better individual classification accuracies. This would decrease the number of motions that a patient would be required to perform, as well as the size of the input data sets. The WE and HO motions provided low ranges of classification accuracies (Table 3), so they were eliminated from the majority vote decision. The RD motion was also removed from the majority vote decision because, with the exception of one classification model, the individual motion models provided classification accuracies below 70%. Majority vote decisions based on the outputs of the top motions (EF, EE, P, S, WF, UD, and HC) were weighted equally.

A weighted majority vote decision was also applied to the individual motion models. Each model was weighted by its respective classification accuracy. For example, when using the LDA classifier with FS1, the weights for each decision model were selected as follows: EF = 62.3, EE = 65.4, etc., based on the classification results found in Table 2. The sum of the weights of the decision models that identified the patient as healthy was determined, as well as the sum of the weights of the models that identified the patient as injured. The highest sum (representing either healthy or injured) was selected as the final weighted majority vote decision. The weighted majority vote classification accuracies ranged from 64.8–77.2%, and the weighted vote provided improvements to the basic majority vote classification accuracy for all models.

### 4.5. Patient Characteristics

Features representing patient characteristics of sex, age, BMI, and time since injury were added to FS1–FS5, and the classification accuracies were obtained using leave-one-patient-out cross-validation. The inclusion of the patient characteristic features did not significantly improve the classification accuracies for any of the motion models. Based on this analysis, the patient characteristics of sex, age, BMI, time since injury, and hand dominance do not provide important information for the classifiers tested that could assist with determining the category of muscle health.

## 5. Discussion

The feature sets recommended in the literature were first explored for classifying muscle health. When compared to FS1, FS2 performed similarly with the various classifiers, but had an overall worse performance than FS1. FS3 was unique in that there was not a single classifier that was the best; however, FS3 also provided the highest accuracies out of all feature sets. These observations are consistent with the literature, which suggests that feature set selection is more important than classifier selection for obtaining high classification accuracy with EMG signals [36].

The addition of more features in the feature set can improve accuracy, until an asymptote is reached, at which point adding new features will not improve the accuracy [13]. The inclusion of four features in FS1 compared to two in FS2 could account for the better classification performance of FS1. Likewise, FS3 contained the greatest number of features of the three feature sets tested, and displayed higher accuracies than FS1 and FS2.

The LDA classifier tended to provide better classification for individual features; however, the RF classifier provided better accuracies when used with feature sets. As the RF classifier can classify based on nonlinear relationships between features, this may have contributed to the higher performance when used with feature sets.

The EE motion was found to provide best classification, and the WE, HO, and RD motions were found to provide poor classification accuracies. The WE, HO, and RD motions are hand and wrist motions, therefore, the performance of these motions may be less impacted by an injury to the elbow. For example, the HO motion involves the relaxation of the forearm muscles as the hand is released from the closed position, which may be less strenuous on the elbow. The lower classification performance may also be due to the muscles involved in the motion. The primary muscles involved in RD, the extensor carpi radialis and the flexor carpi radialis, were not used as inputs for the classification models. The WE motion is primarily performed by the ECU muscle, which suggests that the activation of the ECU muscle is less informative for assessing elbow muscle health.

The RELIEFF algorithm identified the MFL, MYOP, PSR, and MSD features as preferable for identifying muscle health. The feature set FS4 provided the highest classification accuracy achieved (82.1%). Although not ideal, this sets a baseline for future comparisons.

This study used the injured and uninjured limbs of the same participants. This design ensured that the healthy and injured data sets were matched for the population of patients (in terms of age, sex, and BMI) presenting at clinics with elbow trauma injuries. However, this design does not consider potential differences in muscle activity due to handedness. For example, research suggests that biceps activity is lower in the dominant arm [39]. Overuse of the healthy limb to compensate for the loss of function in the injured limb could also have influenced the results.

ROM and strength recovery were not measured in this study. It is recommended that for future studies, a cohort of patients should be observed at multiple stages over the recovery process, to observe healing patterns within individuals.

The purpose of the model was to allow for an objective metric of muscle health to be determined that could identify if a patient was healing. This work was also directed towards identifying trends in EMG behaviour that reflect muscle health following elbow trauma. EMG has been used to study the muscle health of patients with neuromuscular injuries; however, no models have yet been developed to identify and diagnose the muscle health of elbow trauma patients. This is the first model of its kind. There is evidence that this could be a precursor to developing a more advanced model of muscle health, so that a patient could be monitored as they progress through the rehabilitation process.

To develop a practical model of muscle health for a wearable device with multiple classes of patient health, future work should be directed towards collecting data from a larger cohort of patients with similar injuries, or from the same patients at multiple stages of recovery.

## 6. Conclusions

This paper introduced and evaluated a method of using sEMG signals to classify subjects between two levels of upper-limb muscle health. The models developed achieved classification accuracies of 45.9–82.1%. The healthy and injured data sets were collected from the same patient, so that the healthy data sets can be compared to the injured sets, and can allow for a better representation of the population of patients (in terms of age, sex, BMI) presenting at clinics with elbow trauma injuries. EMG features capable of predicting muscle health were identified. The best individual features were identified to be MFL, MYOP, PSR, and spike shape analysis features, in particular MSD. The best individual motions for classifying health were EF, EE, P, S, WF, UD, and HC. The first classification models to distinguish between healthy and injured limbs of elbow trauma patients based on EMG data were developed. There is the potential for implementing a classification model of health in a rehabilitative elbow brace to assess patients recovering from elbow trauma; however, further work in this direction, including further data collection, validation, optimization, and improvements to the existing state-of-the-art EMG acquisition systems will be necessary to achieve this goal.

## Figures and Tables

**Table 1:** List of common sEMG features with references. *K* is the number of window segments used for multi-window features.

Feature	Abbr.	Reference	Feature	Abbr.	Reference
Average amplitude change	AAC	[13]	Mean spike amplitude	MSA	[14]
Autoregressive coefficients (second and fourth order)	AR2 AR4	[13,15]	Mean spike duration	MSD	[14]
Approximate entropy	ApEn	[12,16]	Mean spike frequency	MSF	[14]
Coefficients of cepstral analysis (fourth order)	CC4	[13]	Mean spike slope	MSS	[14]
Difference absolute standard deviation value	DASDV	[13]	Multiple trapezoidal windows (K=3)	MTW	[12]
Detrended fluctuation analysis	DFA	[17]	Myopulse percentage rate	MYOP	[13]
Frequency ratio	FR	[13]	Peak frequency	PKF	[13]
Higuchi’s fractal dimension	HFD	[17]	Power spectrum ratio	PSR	[13]
Kurtosis	KURT	[12]	Root mean square	RMS	[13]
Log detector	LOG	[13]	Skewness	SKEW	[12]
Mean absolute value	MAV	[18]	Spectral moments	SM1 SM2 SM3	[13]
Mean absolute value slope (K=3)	MAVS	[18]	Slope sign change	SSC	[18]
Median frequency	MDF	[13]	Sample entropy	SampleEn	[12,19]
Maximum fractal length	MFL	[17]	Total power	TTP	[13]
Multiple Hamming windows (K=3)	MHW	[12]	Variance of EMG	VAR	[13]
Modified mean absolute value 1	MMAV1	[13]	Variance of central frequency	VCF	[13]
Modified mean absolute value 2	MMAV2	[13]	Willison amplitude	WAMP	[13]
Mean frequency	MNF	[13]	Waveform length	WL	[18]
Mean power	MNP	[13]	Zero crossings	ZC	[18]
Mean number of peaks per spike	MNPPS	[14]			

**Table 2:** Classification accuracies for each preliminary feature set. The best classification result for each motion within each feature set is in bold.

Feature Set	Motions	Classification Accuracy (%)		
		LDA	SVM	RF
FS1 (MAV, SSC, WL, ZC)	EF	62.3	60.5	**70.3**
	EE	65.4	62.3	**71.6**
	P	**69.1**	67.9	67.3
	S	60.5	62.3	**68.5**
	WF	67.3	56.8	**69.1**
	WE	55.6	**64.8**	56.8
	UD	58.6	62.3	**71.6**
	RD	61.7	65.4	**66.0**
	HC	64.8	54.9	**72.2**
	HO	55.6	**61.1**	57.4
FS2 (RMS, AR2)	EF	59.9	57.4	**67.9**
	EE	61.7	64.8	**69.8**
	P	67.3	49.4	**71.6**
	S	58.6	54.9	**69.8**
	WF	63.6	59.3	**65.4**
	WE	**60.5**	54.3	59.9
	UD	62.3	57.4	**63.0**
	RD	59.1	45.9	**69.2**
	HC	63.0	56.2	**66.7**
	HO	58.6	**64.2**	63.0
FS3 (MSA, MSF, MSS, MNPPS, MSD)	EF	**74.1**	61.1	64.8
	EE	61.1	**77.8**	68.5
	P	**72.2**	63.0	61.1
	S	50.0	63.0	**72.2**
	WF	**79.6**	64.8	75.9
	WE	57.4	**66.7**	64.8
	UD	**72.2**	68.5	61.1
	RD	61.1	**77.8**	57.4
	HC	57.4	59.3	**64.8**
	HO	48.2	50.0	**51.9**

**Table 3:** Range of classification accuracies for each motion.

Motion	Classification Accuracy (%)
EF	57.4–74.2
EE	62.3–77.8
P	49.4–72.2
S	50.0–72.2
WF	56.8–79.6
WE	54.3–66.7
UD	57.4–72.2
RD	45.9–77.8
HC	54.9–72.2
HO	48.2–64.2

**Table 4:** Majority vote classification accuracies for individual features. Features are ordered by LDA classification accuracy. The best classifier result for each feature is in bold.

Feature	Classification Accuracy (%)			Feature	Classification Accuracy (%)		
	LDA	SVM	RF		LDA	SVM	RF
MFL	**76.54**	73.45	59.88	MMAV1	**64.81**	54.94	62.35
MYOP	**74.69**	66.67	58.64	HFD	**64.20**	62.35	59.88
MSD	**74.69**	54.94	55.56	MAVS	**64.20**	50.00	56.17
AR4	**74.07**	59.88	50.00	PKF	63.58	**67.9**	61.11
MSF	**72.84**	**72.84**	54.94	MAV	63.58	55.60	**64.81**
MNPPS	**70.99**	64.20	52.47	MSA	**63.58**	53.70	62.35
PSR	**70.99**	66.67	56.17	MTW	62.96	50.62	**64.20**
ApEn	**69.14**	65.43	57.41	RMS	62.35	57.41	**65.43**
LOG	**69.14**	57.41	63.58	MHW	61.73	51.85	**62.34**
MNF	**69.14**	68.52	54.32	SM3	60.49	**61.73**	60.49
ZC	**68.52**	62.35	55.56	MNP	59.88	52.47	**63.58**
DASDV	**68.52**	51.85	61.73	TTP	58.79	51.85	**64.20**
VCF	**68.52**	57.41	56.17	VAR	58.64	52.47	**64.20**
AAC	**67.90**	51.85	59.88	FR	58.02	**67.90**	58.02
MSS	**67.90**	51.85	56.17	SM1	58.02	51.85	**61.11**
MMAV2	**67.38**	54.32	61.73	SKEW	**57.41**	53.09	50.00
WL	**66.05**	51.85	56.17	DFA	**56.80**	46.30	50.00
CC4	**66.05**	51.23	50.62	SM2	55.56	56.79	**59.23**
MDF	**65.43**	**65.43**	56.79	WAMP	54.32	**56.17**	50.62
SampleEn	**65.43**	64.81	56.17	KURT	52.47	**53.70**	50.00
SSC	**64.81**	61.73	51.85				

**Table 5:** Classification accuracies for each feature set. The best classification result for each motion within each feature set is in bold.

Feature Set	Motions	Classification Accuracy (%)		
		LDA	SVM	RF
FS4 (MFL, MYOP)	EF	**78.4**	70.4	63.6
	EE	**68.5**	65.4	67.3
	P	**71.6**	70.4	63.6
	S	**72.2**	71.0	70.4
	WF	70.3	**70.4**	**70.4**
	WE	**66.0**	60.5	57.4
	UD	77.2	**79.6**	67.3
	RD	**74.7**	69.1	67.9
	HC	**69.8**	**69.8**	63.6
	HO	63.0	**70.4**	63.6
FS5 (LOG, DASDV, MYOP, MAVS, PSR, AR4, ApEn, MFL, MSD)	EF	61.7	**73.5**	71.0
	EE	**75.9**	72.8	72.8
	P	59.9	66.7	**67.9**
	S	58.0	**71.0**	67.9
	WF	64.8	59.9	**69.1**
	WE	51.9	58.6	**63.0**
	UD	69.1	**74.7**	64.8
	RD	61.1	**73.5**	67.3
	HC	56.2	63.6	**67.3**
	HO	56.8	**64.8**	60.5
FS5 Optimized with RELIEFF (PSR, MFL, MSD)	EF	69.8	**71.6**	64.8
	EE	72.5	72.2	**75.9**
	P	**72.8**	70.4	66.0
	S	71.0	**72.8**	70.4
	WF	56.8	**69.1**	68.5
	WE	61.7	**66.7**	64.2
	UD	72.2	**76.5**	69.1
	RD	67.3	64.8	**68.5**
	HC	65.4	**71.0**	61.7
	HO	63.6	66.7	**71.6**

**Table 6:** Majority vote classification accuracies. Majority vote decisions were developed from all ten motions, from only the top motions (EF, EE, P, S, WF, UD, and HC), and from a weighted majority vote. The best classification results within each feature set are in bold.

Feature Set	Motions	Classification Accuracy (%)		
		LDA	SVM	RF
FS1	All	67.9	69.8	**71.0**
	Top	70.4	69.1	**75.3**
	Weighted	72.2	71.0	**74.1**
FS2	All	**70.4**	58.6	69.8
	Top	**69.1**	60.5	67.9
	Weighted	73.5	64.8	**75.3**
FS3	All	71.6	**74.7**	71.6
	Top	72.2	72.8	**74.1**
	Weighted	71.6	73.5	**77.2**
FS4	All	**77.8**	73.4	62.3
	Top	**79.6**	77.8	64.2
	Weighted	**82.1**	74.1	71.0
FS5	All	64.8	**73.5**	66.7
	Top	68.5	**76.5**	65.4
	Weighted	67.3	**75.9**	75.3
FS5 Optimized with RELIEFF	All	74.1	**74.8**	63.0
	Top	74.1	**77.2**	62.3
	Weighted	79.6	**81.5**	77.2
